# Boosting Stability of Hybrid Manganese Bromide Microcrystals by Zn^2+^ Substitution for Wide-Gamut Displays

**DOI:** 10.3390/ma19173650

**Published:** 2026-08-27

**Authors:** Huidong Tang, Pengcheng Jiang, Xinyi Wen, Yulu He, Simeng Wu, Xin Xiong, Yanqiao Xu, Zhi Wu, Qing Hu

**Affiliations:** 1 School of Material Science and Engineering, Hunan Institute of Technology, Hengyang 421002, Chinawenxinyi0410@163.com (X.W.); 15573586300@163.com (Y.H.); 18244861189@163.com (S.W.);; 2 National Engineering Research Center for Domestic & Building Ceramics, Jingdezhen Ceramic University, Jingdezhen 333001, China; xuyanqiao@jcu.edu.cn; 3 School of Material Science and Engineering, Jingdezhen Ceramic University, Jingdezhen 333001, China

**Keywords:** Zn^2+^-substituted hybrid manganese bromide, narrow-band green emitters, stability, wide-gamut displays

## Abstract

Hybrid manganese bromides have emerged as promising lead-free narrow-band green emitters for wide-gamut displays, but their practical application is still limited by insufficient operational stability. Herein, Zn^2+^ substitution is employed to enhance the stability of tetraethylammonium manganese bromide (TEA_2_MnBr_4_) microcrystals (MCs) synthesized via an ultrafast self-assembly method. With increasing Zn^2+^ contents, the photoluminescence intensity of TEA_2_Mn_1−*x*_Zn*_x_*Br_4_ MCs gradually decreases, while the *x* = 0.10 MCs still exhibit efficient narrow-band green emission at 518 nm and a high photoluminescence efficiency of about 85% under blue excitation. More importantly, Zn^2+^ incorporation substantially enhances the storage and thermal stability, as well as operational stability of the corresponding green LED devices (retaining above 99% of initial luminous efficacy after 160 h continuous operation at 20 mA). Finally, a white LED fabricated using the *x* = 0.10 MCs achieves a high luminous efficiency of about 136 lm/W and covers about 112% of the National Television System Committee 1931 color gamut. These results demonstrate that Zn^2+^ substitution is an effective strategy for stabilizing hybrid manganese bromide emitters and highlight their potential for lead-free wide-gamut display applications.

## 1. Introduction

Wide-gamut displays have become a central direction for next-generation liquid-crystal displays and mini-/micro-light-emitting diode (LED) technologies, where highly efficient and stable red, green, and blue emitters are essential for improving color saturation, brightness, efficiency, and operating life [1]. Among the three primary colors, narrow-band green emission plays a particularly critical role, as both its spectral position and full width at half maximum (FWHM) determine the attainable color gamut [2]. Commercial green phosphors, such as β-SiAlON:Eu^2+^, have been widely used in display backlights, but their relatively broad emission and limited spectral tunability restrict further expansion of the display color gamut [3,4]. Although lead-halide perovskites present high color purity and solution processability, their practical application remains hindered by lead toxicity, moisture/thermal instability, and device degradation under long-term operation [5,6]. Therefore, developing lead-free, efficient, and stable narrow-band green emitters remains an important challenge for advanced display applications.

Hybrid manganese halides with unique isolated [MnX_4_]^2−^ tetrahedra have recently attracted considerable attention as narrow-band green emitters owing to their low toxicity, low-cost solution processability, and excellent fluorescence properties [7,8,9]. For instance, Yu et al. [10] reported (4-BTP)_2_MnBr_4_ (4-BTP = (4-bromobenzyl)triphenylphosphonium) with a green emission at 524 nm and a near-unity photoluminescence quantum yield (PLQY), resulting in the prepared white LED device with a wide color gamut of 100.7% National Television System Committee (NTSC) 1931. Zhang et al. [11] developed (MTP)_2_MnBr_4_ single crystals, achieving a 516 nm green emission with a FWHM of 44 nm and a PLQY of 99.5%. The fabricated white LED has a luminous efficiency (LE) of 101 lm/W, a wide color gamut of 116 % NTSC 1931, and good operational stability (maintaining LE of 95 lm/W after working for 24 h). Despite the high PLQYs achieved in hybrid manganese halides, their long-term environmental and operational stability remains an important challenge.

Zero-dimensional hybrid manganese halides generally consist of isolated metal–halide units separated by organic cations, and their structural stability depends on electrostatic interactions, hydrogen bonding, and other relatively weak interactions between the organic and inorganic components [8,12,13]. To overcome the above challenges, organic cationic strategies have been developed. For instance, Jiang et al. [14] employed tetraethylammonium (TEA^+^) as the organic cation to construct zero-dimensional (TEA)_2_MnBr_4_ single crystals, resulting in narrow-band green emission at 515 nm and a high PLQY of 85.1%. These single crystals also exhibited good storage and thermal stability, with an essentially unchanged PLQY after one month of storage in air and retention of 63% of the initial PL intensity at 130 °C. The as-fabricated WLED exhibited LE of 96 lm/W, 104% NTSC 1931 and good operational stability (almost unchanged PL intensity and chromaticity coordinate of the WLED after 3000 h operational time). In addition, metal-site ion substitution, such as Zn^2+^ incorporation, provides an effective strategy for improving stability of hybrid manganese halide emitters [15,16,17]. For example, Zhao et al. [18] reported that (C_13_H_26_N)_2_Zn_0.2_Mn_0.8_Br_4_ exhibited markedly enhanced stability (85% retention of the room-temperature PL intensity at 420 K) of the fabricated white LED with a wide color gamut of 112.2% NTSC. Pan et al. [19] demonstrated that Zn alloying in [(CH_3_)_4_N]_2_Mn_0.6_Zn_0.4_Cl_4_ induced remarkable anti-thermal-quenching behavior, with the PL intensity at 400 K enhanced by 40% relative to room temperature. Yan et al. [17] developed Zn^2+^-doped (C_12_H_28_N)_2_MnBr_4_ with good environmental stability and fabricated a WLED with good operational stability with essentially unchanged PL spectra and luminance of backlight display device after 48 h operation. These impressive results reveal that Zn substitution can regulate the local coordination environment, inhibit ion migration and increase the thermal activation energy, thereby improving structural and operational stability. For (TEA)_2_MnBr_4_, Wang et al. [20] adopted a Zn-rich host strategy and prepared sub-micrometer (TEA)_2_Mn_0.7_Zn_0.3_Br_4_ phosphors with green emission at 517 nm, FWHM of 47 nm, and a PLQY of 86.6% under 450 nm excitation. The (TEA)_2_Mn_0.7_Zn_0.3_Br_4_ phosphors have good thermal stability compared with (TEA)_2_MnBr_4_. When combined with K_2_SiF_6_:Mn^4+^ red phosphors, the fabricated WLED device has 101% NTSC and unchanged PL intensity after 300 min of operation at 15 mA. Yang et al. [21] introduced 5 mol% Zn^2+^ into (TEA)_2_MnBr_4_ single crystals. The optimized (TEA)_2_Mn_0.95_Zn_0.05_Br_4_ MCs exhibit an increased PLQY of 84.3%, compared with 81.7% for the undoped sample. However, the facile and rapid synthesis of highly efficient and stable Zn-doped TEA_2_MnBr_4_ microcrystals (MCs), together with a systematic investigation of their device operational stability, remains challenging.

Herein, we report a facile ultrafast self-assembly strategy for preparing Zn^2+^-substituted TEA_2_Mn_1−x_Zn_x_Br_4_ microcrystals (MCs) as narrow-band green emitters for wide-gamut displays. The structure, morphology, PL properties, and stability of TEA_2_Mn_1−x_Zn_x_Br_4_ MCs were studied. The as-prepared *x* = 0.10 MCs exhibit strong green emission at 518 nm under blue excitation with a high PLQY of about 85%. The storage and thermal stability of the MCs, as well as the operational stability of the corresponding green LED devices, were systematically investigated. The green LED based on the *x* = 0.10 MCs can retain above 99% of initial LE after continuous operation at 20 mA for 160 h. Finally, a white LED fabricated using *x* = 0.10 MCs and K_2_SiF_6_:Mn^4+^ red phosphors achieves a high LE and a wide color gamut, demonstrating the potential of Zn^2+^-substituted hybrid manganese bromides for stable, lead-free, and wide-gamut display applications.

## 2. Materials and Methods

### 2.1. Materials

Manganese (II) bromide tetrahydrate (MnBr_2_·4H_2_O, 99%, Aladdin, Shanghai, China), zinc bromide (ZnBr_2_, 99.9%, Aladdin), tetraethylammonium bromide ((C_2_H_5_)_4_NBr, TEABr, 98%, Aladdin), and ethanol (C_2_H_5_OH, ≥99.7%, Sinopharm, Beijing, China) were used without further purification.

### 2.2. Synthesis of TEA_2_Mn_1−x_Zn_x_Br_4_ MCs via an Ultrafast Self-Assembly Method

The TEA_2_Mn_1−x_Zn_x_Br_4_ (x = 0.00, 0.05, 0.10, 0.15, 0.20 and 0.25) MCs were synthesized by an ultrafast self-assembly method. For the sample with *x* = 0.00, TEABr (2.25 mmol) and MnBr_2_·4H_2_O (1 mmol) were added in alcohol (10 mL) under stirring at 500 rpm. A green suspension rapidly formed within 10 s at room temperature. To ensure complete reaction, the suspension was further stirred for 2 h. The green suspension was washed three times with ethanol and collected by centrifugation at 1000 rpm. The resulting TEA_2_MnBr_4_ MCs were obtained after drying in a conventional oven at 60 °C for 6 h. For the solid-solution sample (x = 0.05, 0.10, 0.15, 0.20 and 0.25), the same synthesis procedure was followed, except that MnBr_2_·4H_2_O was partially replaced by the corresponding amount of ZnBr_2_. The detailed nominal compositions and precursor amounts are summarized in Table S1. The yield is above 87%.

### 2.3. Fabrication of LED Devices

To fabricate green-emitting LEDs, *x* = 0.00 MCs or *x* = 0.10 MCs and UV-curable silicone (DOWSIL 3-6371) were thoroughly mixed at a mass ratio of 1:2 (MCs:silicone). The resulting mixture was coated onto a 450 nm blue InGaN chip and cured under UV irradiation for 15 min. For WLED fabrication, *x* = 0.10 MCs and K_2_SiF_6_:Mn^4+^ red phosphors were homogeneously dispersed in UV-curable silicone at a mass ratio of 1:0.8:2 (green MCs:red phosphor:silicone). The mixture was then deposited onto a 450 nm blue InGaN chip and cured under UV irradiation for 15 min to obtain the white LED device.

### 2.4. Characterization

The powder X-ray diffraction (XRD) patterns of TEA_2_Mn_1−*x*_Zn*_x_*Br_4_ MCs were recorded with an X-ray diffractometer (Bruker D8 Advance, Billerica, MA, USA) with Cu Kα radiation (*λ* = 1.5406 Å), scan range of 5–90°, step size 0.02°, and scan rate 10°/min. Scanning electron microscopy (SEM, ZEISS Gemini 300, Oberkochen, Germany) coupled with energy-dispersive X-ray spectroscopy (EDS) was used to examine the surface morphology and spatial elemental distribution of Zn^2+^-substituted TEA_2_MnBr_4_ MCs. The chemical states of the constituent elements were probed by X-ray photoelectron spectroscopy (XPS, Thermo Scientific K-Alpha, East Grinstead, UK) using a monochromated Al Kα X-ray source with an excitation energy of 1486.68 eV. The high-resolution XPS spectra were acquired with an energy step of 0.1 eV. PL excitation (PLE) spectra, PL spectra, PL decay, and temperature-dependent PL spectra of TEA_2_Mn_1−*x*_Zn*_x_*Br_4_ MCs were recorded using a fluorescence spectrometer (Edinburgh FS5 v2, Scotland, UK). The photoluminescence quantum yield (PLQY), defined as the ratio of the number of emitted photons to the number of absorbed excitation photons, and the absorption efficiency (*η*_ab_), defined as the fraction of incident excitation photons absorbed by the sample, were measured using the FS5 spectrofluorometer equipped with an integrating sphere under direct excitation at 455 nm. The reference and sample spectra were collected using the same sphere geometry, powder holder, sample-loading procedure, and instrumental settings. The PLQY and *η*_ab_ values were directly obtained using Fluoracle 3.0 software. The uncertainties represent the standard deviation of *n* = 3 replicate measurements. Thermogravimetric (TG) analysis was performed using a NETZSCH 309 thermogravimetric analyzer (NETZSCH, Selb, Germany) under an Ar atmosphere over the temperature range of 25–600 °C at a heating rate of 10 °C/min. The electroluminescence (EL) spectra and operational stability of the LED devices were characterized by an HP8000 spectroradiometer (Hopocolor, Hangzhou, China). The CIE 1931 chromaticity coordinates, color rendering index (CRI), correlated color temperature (CCT), luminous flux, and luminous efficacy (LE) were directly obtained using the calibrated HP8000 analysis software V2.1. The CIE chromaticity coordinates describe the color of the emitted light in the CIE 1931 x–y chromaticity diagram, whereas the CRI characterizes the color-rendering ability of a light source. LE was defined as the luminous flux divided by the electrical input power of the LED and is expressed in lm/W. The color-gamut coverage was calculated from the chromaticity coordinates of the red, green, and blue primaries as the ratio of the corresponding color-triangle area to that of the NTSC reference triangle in the CIE 1931 chromaticity diagram. The operational stability of the green LED devices was evaluated under continuous operation at a constant driving current of 20 mA at 25 °C and 50% relative humidity. The devices remained continuously powered throughout the aging experiment. Luminous efficacy and EL spectra were recorded at intervals of 10 min. Two independently fabricated green LED devices were tested for each composition (*n* = 2) under continuous operation at 20 mA.

## 3. Results and Discussion

**Figure 1:**
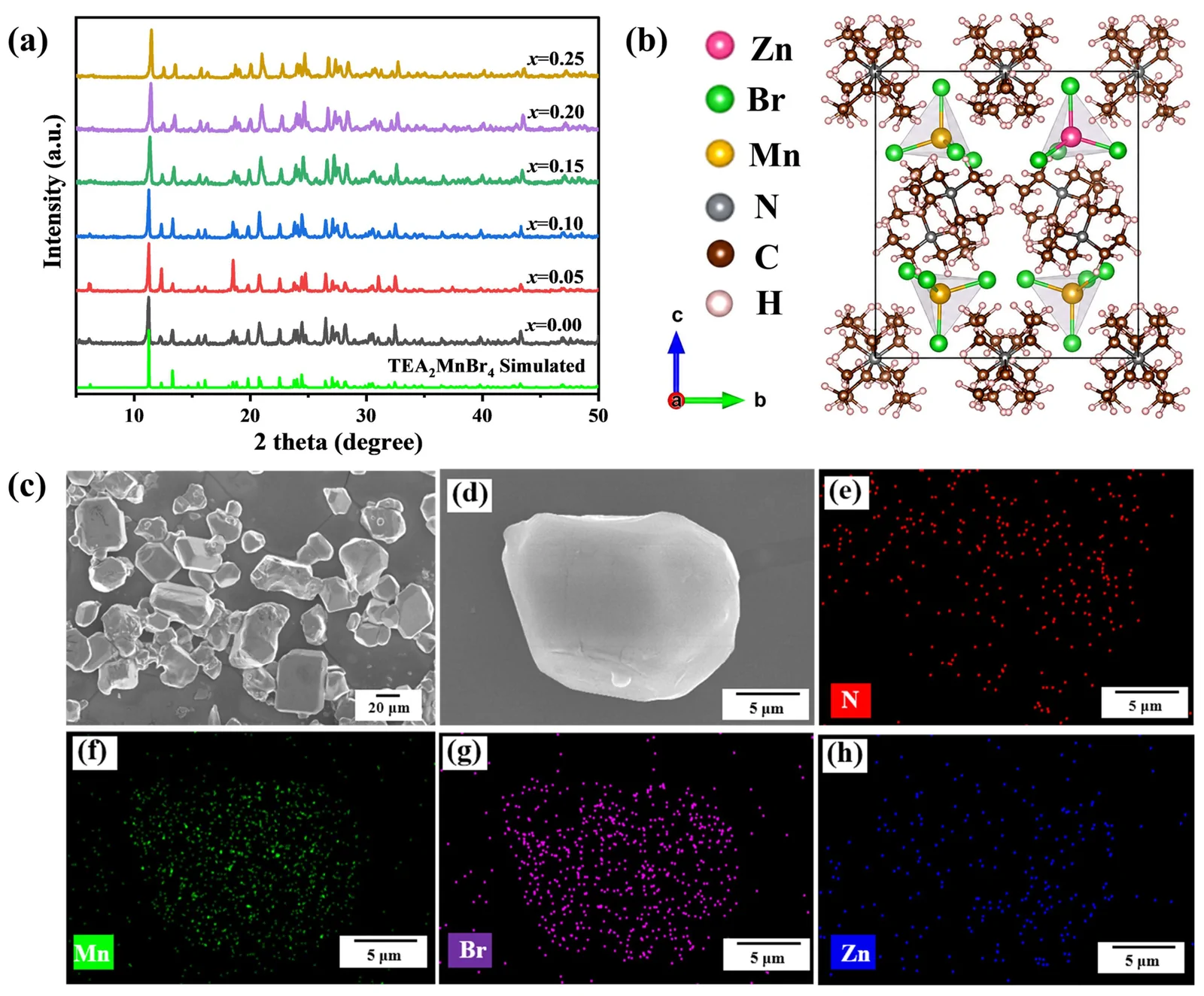
(**a**) XRD patterns of nominal TEA_2_Mn_1−*x*_Zn*_x_*Br_4_ MCs prepared with different Zn precursor fractions, (**b**) crystal structure, (**c**) low-magnification SEM image and (**d**) higher-magnification SEM image of a *x* = 0.10 MCs, and corresponding EDS elemental maps of (**e**) N, (**f**) Mn, (**g**) Br, and (**h**) Zn.

A series of TEA_2_Mn_1−*x*_Zn*_x_*Br_4_ MCs with different nominal Zn^2+^ substitution amounts were prepared via an ultrafast self-assembly method (Table S1). The excess TEABr is used to suppress the formation of bromine vacancies and enhance radiative recombination of MCs [22]. Figure 1a shows the XRD patterns of the TEA_2_Mn_1−*x*_Zn*_x_*Br_4_ MCs synthesized with different Zn^2+^ substitution concentrations. The diffraction peaks of the samples synthesized by the ultrafast self-assembly method are in good agreement with the simulated pattern of TEA_2_MnBr_4_ based on the crystallographic data of Cambridge Crystallographic Data Centre (CCDC) 2042083 [14]. As the Zn^2+^ substitution concentration increases from *x* = 0 to *x* = 0.25, the diffraction peaks show a slight shift toward higher angles, which can be attributed to the smaller effective ionic radius of Zn^2+^ (0.60 Å) compared with that of Mn^2+^ (0.66 Å), leading to lattice contraction after the partial substitution of Mn^2+^ by Zn^2+^ [23]. The XRD Rietveld refinements were performed for the samples with *x* = 0.00, 0.10, and 0.25, as shown in Figure S1 and Table S2. In the crystal structure, TEA_2_Mn_1−*x*_Zn*_x_*Br_4_ has the tetragonal $P\bar{4}2_{1}m$ space group (No. 113), in which the [MnBr_4_]^2−^ unit is replaced by [ZnBr_4_]^2−^. The [MnBr_4_]^2−^ and [ZnBr_4_]^2−^ units are isolated and separated by TEA^+^ ions (Figure 1b). With increasing nominal Zn content from 0 to 0.25, *a* = *b* decreases from approximately 13.3810(4) to 13.3655(2) Å, c decreases from 14.4200(4) to 14.4083(4) Å, and the unit-cell volume (*V*) decreases from 2581.92(16) to 2573.84(11) Å^3^. The linear decrease in the lattice parameters is consistent with Vegard-like behavior over the investigated composition range. The XRD results confirm the successful incorporation of Zn^2+^ into the TEA_2_MnBr_4_ lattice, yielding TEA_2_Mn_1−*x*_Zn*_x_*Br_4_ solid solutions. Figure 1c,d show the SEM image and EDS elemental mapping of the *x* = 0.10 MCs. The obtained MCs exhibit irregular particle morphology. Zn is present and spatially distributed together with Mn and Br in the analyzed particle. Semi-quantitative EDS analysis gives a Zn/(Zn + Mn) ratio of 9.7%, which is close to the nominal value of 10% (Table S3). These results demonstrate the successful incorporation of Zn^2+^ into the TEA_2_MnBr_4_ lattice via the ultrafast self-assembly method.

**Figure 2:**
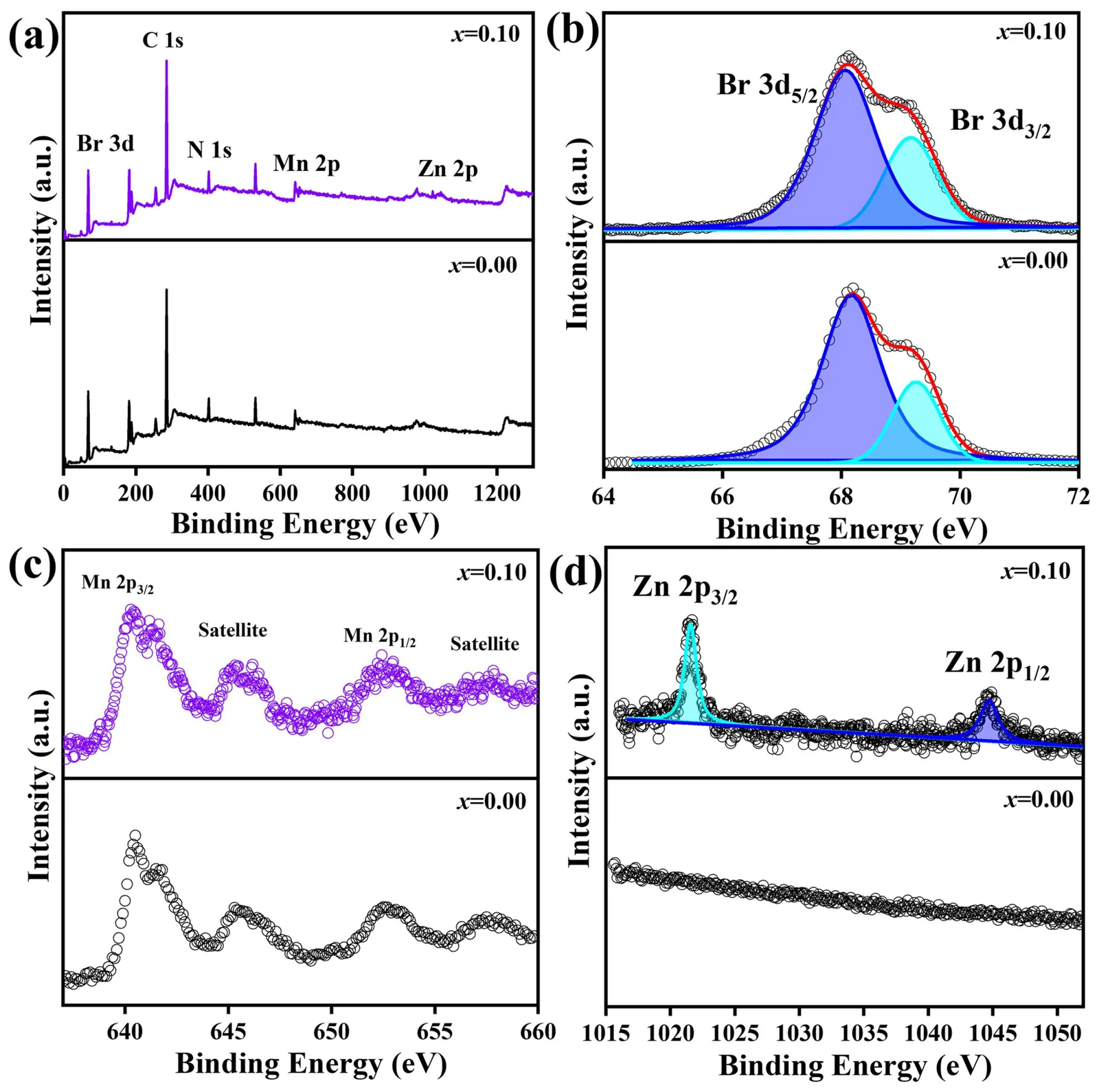
(**a**) XPS survey spectra and HR-XPS spectra of (**b**) Br 3d, (**c**) Mn 2p, and (**d**) Zn 2p for *x* = 0.00 and *x* = 0.10 MCs.

Figure 2 shows the XPS spectra and high-resolution XPS spectra (HR-XPS) of *x* = 0.00 and *x* = 0.10 MCs. As shown in the survey spectra in Figure 2a, Mn 2p and Br 3d signals are detected in the unsubstituted MCs, whereas Mn 2p, Zn 2p, and Br 3d signals are observed in the *x* = 0.10 MCs. In addition, the surface-sensitive and semi-quantitative XPS analysis gives a Zn/(Zn + Mn) ratio of 9.3%, which is close to the EDS result and the nominal value of 10% (Table S4). As shown in Figure 2b, the high-resolution Br 3d spectra exhibit a spin–orbit doublet at approximately 68.2 and 69.3 eV, assigned to Br 3d_5/2_ and Br 3d_3/2_, respectively. The high-resolution Mn 2p spectra show complex multiplet-split envelopes, with principal features at approximately 640.2 and 652.6 eV in the Mn 2p_3/2_ and Mn 2p_1/2_ regions (Figure 2c), respectively. The higher-binding-energy features at approximately 645.6 and 657.4 eV are attributed to multiplet and satellite contributions characteristic of high-spin Mn^2+^ [16,20]. Moreover, two distinct Zn 2p signals located at 1021.6 and 1044.7 eV are assigned to Zn 2p_3/2_ and Zn 2p_1/2_ (Figure 2d), respectively [21], confirming the successful incorporation of Zn^2+^ into the TEA_2_MnBr_4_ crystal lattice.

**Figure 3:**
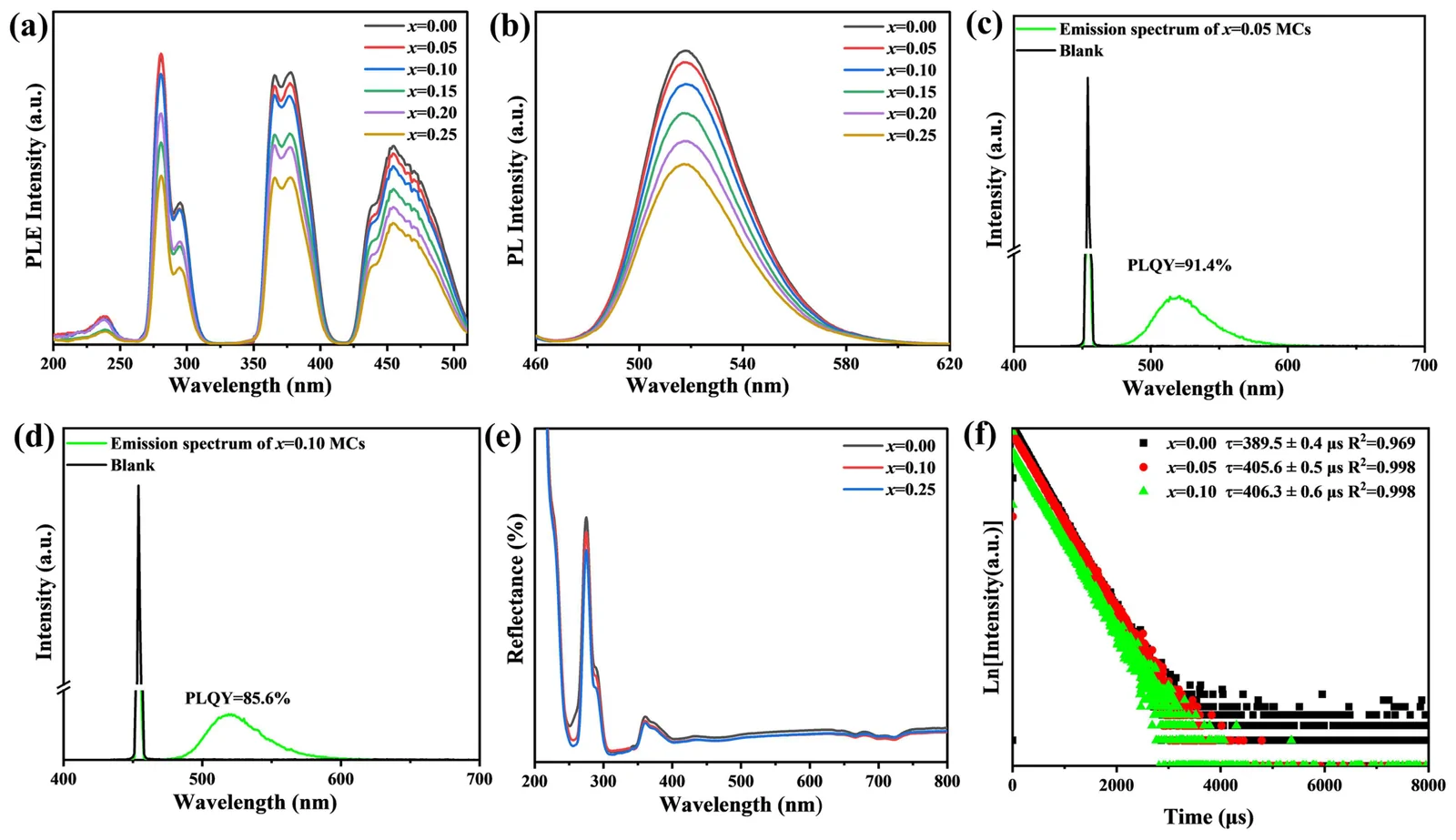
(**a**) PLE spectra monitored at 518 nm, (**b**) PL spectra of TEA_2_Mn_1−*x*_Zn*_x_*Br_4_ MCs with different Zn substitution contents recorded under 455 nm excitation, (**c**) PLQY of *x* = 0.05 MCs, (**d**) PLQY of *x* = 0.10 MCs, (**e**) diffuse-reflectance spectra, and (**f**) PL decay of TEA_2_Mn_1−*x*_Zn*_x_*Br_4_ MCs with different Zn substitution contents.

Figure 3a shows the PLE spectra of TEA_2_Mn_1−*x*_Zn*_x_*Br_4_ MCs with different Zn^2+^ contents. All samples exhibit similar excitation profiles when monitored at 518 nm. The excitation peaks at approximately 361, 375, 430, 455, and 470 nm can be assigned to the ^6^A_1_(S) → ^4^E(D), ^6^A_1_(S) → ^4^T_2_(D), ^6^A_1_(S) → ^4^A_1_/^4^E(G), ^6^A_1_(S) → ^4^T_2_(G), and ^6^A_1_(S) → ^4^T_1_(G) transitions of tetrahedrally coordinated Mn^2+^, respectively. As Zn^2+^ concentration increases, the excitation intensity of TEA_2_Mn_1−*x*_Zn*_x_*Br_4_ MCs gradually decreases. Under 455 nm radiation, TEA_2_Mn_1−*x*_Zn*_x_*Br_4_ MCs have strong green emission at 518 nm (Figure 3b). No obvious shift in the emission peak is observed upon Zn^2+^ substitution, whereas the PL intensity decreases progressively as the Zn^2+^ content increases, which is in agreement with previously reported results [21]. The gradual decrease in PL intensity at high Zn^2+^ substitution contents may originate from the dilution of Mn^2+^ luminescent centers and possible defect-assisted non-radiative relaxation [20].

The PLQYs of TEA_2_Mn_1−*x*_Zn*_x_*Br_4_ MCs were measured, as shown in Figure 3c,d. Under 455 nm excitation, the *x* = 0.00 MCs exhibit a PLQY of 98.1 ± 0.6%, which is higher than the previously reported values of 85.1% and 81.7% for TEA_2_MnBr_4_ [14,21]. The higher PLQY may be mainly associated with the use of excess TEABr precursor during synthesis, which can suppress the formation of Br vacancies and thereby reduce defect-related non-radiative recombination [22]. As the Zn^2+^ fraction increases from *x* = 0.05 to *x* = 0.25, the PLQY gradually declines, while the *x* = 0.10 MCs possess a PLQY of 85.6 ± 0.6%. As the Zn^2+^ content increases from *x* = 0.00 to *x* = 0.10, the *η*_ab_ increases from 45.0 ± 0.7% to 65.0 ± 1.1%. However, at *x* = 0.25, *η*_ab_ decreases to 43.0 ± 0.6% (Table S5). The diffuse-reflectance spectra of the *x* = 0.00, 0.10, and 0.25 samples exhibit similar spectral features without an obvious new absorption band or pronounced shift in the absorption edge (Figure 3e). The external quantum efficiency (EQE) can be calculated by the formula

EQE = PLQY × *η*_ab_ (1)

The EQE of *x* = 0.10 MCs is 55.6 ± 1.0%. These results show that the PLQY decreases with increasing Zn^2+^ content, whereas *η*_ab_ exhibits a non-monotonic composition dependence, resulting in corresponding variations in EQE.

Figure 3f shows the PL decay curves of TEA_2_Mn_1−*x*_Zn*_x_*Br_4_ MCs with different Zn^2+^ substitution concentrations. These decay curves can be fitted by a single-exponential function, as described by

*I*(t) = *I*_bg_ + A exp(−t/τ) (2)

where *I*_bg_ is the constant background, A is the initial decay amplitude above the background, and τ is the characteristic 1/e lifetime. The decay profiles can be well fitted using a single-exponential function, indicating that the green emission mainly originates from the intrinsic Mn^2+^ luminescent centers in the phosphor lattice. The calculated lifetimes of *x* = 0.00 MCs, *x* = 0.05 MCs, and *x* = 0.10 MCs are 389.5 ± 0.4, 405.6 ± 0.5, and 406.3 ± 0.6 μs, respectively. These microsecond-scale lifetimes are characteristic of the spin-forbidden d–d transition of Mn^2+^. The slight prolongation of PL lifetime after Zn^2+^ incorporation suggests that Zn^2+^ substitution modifies the excited-state relaxation dynamics of Mn^2+^ centers, possibly owing to the altered local coordination environment.

**Figure 4:**
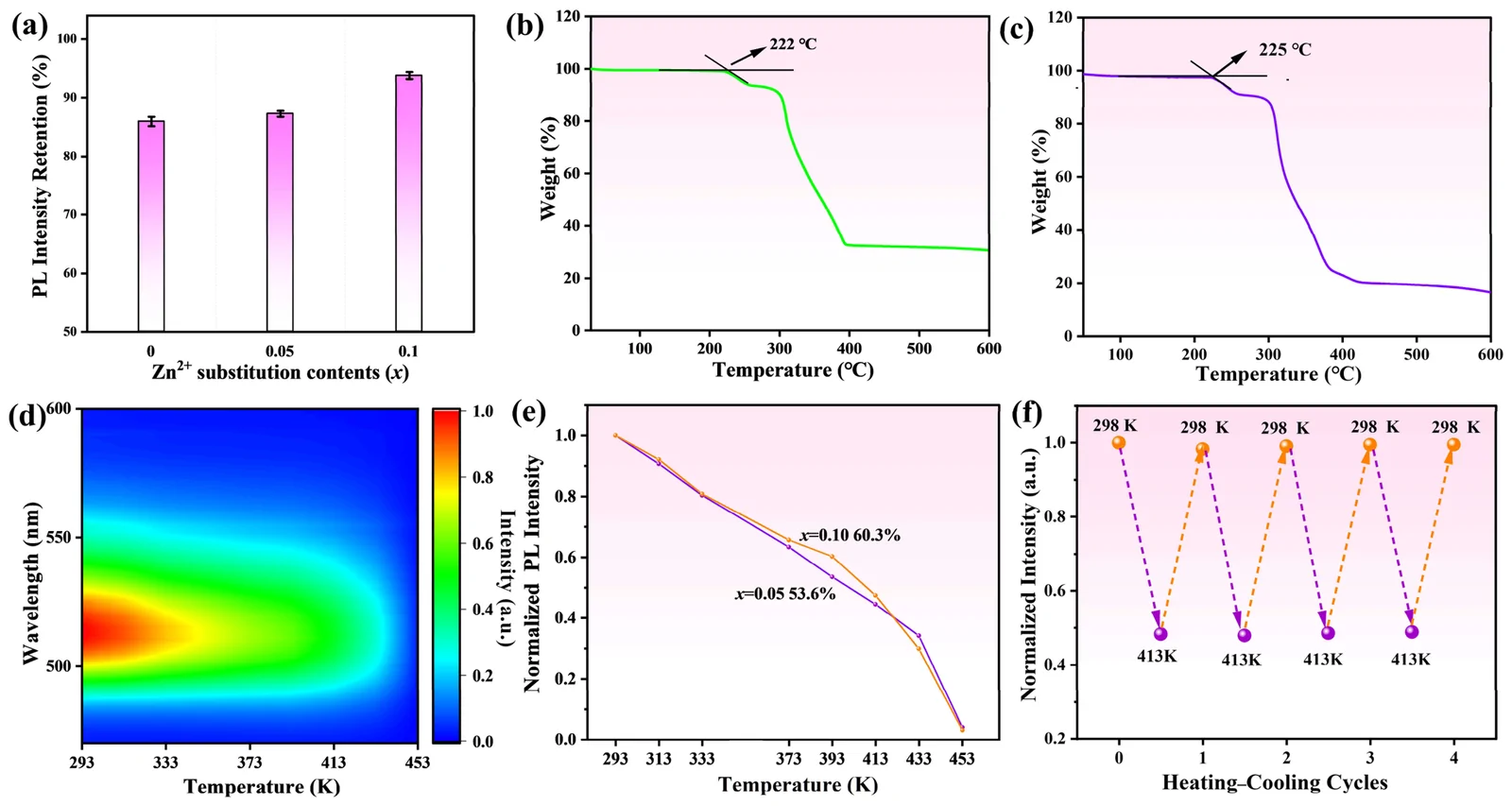
(**a**) Storage stability of TEA_2_Mn_1−x_Zn_x_Br_4_ MCs with different Zn^2+^ substitution contents. Data are presented as mean ± standard deviation from two samples (*n* = 2) for each composition. TG curve of (**b**) *x* = 0.00 and (**c**) *x* = 0.10 MCs, (**d**) temperature-dependent PL emission pseudocolor contour maps of *x* = 0.10 MCs, (**e**) normalized PL intensity vs. temperature of *x* = 0.05 MCs and *x* = 0.10 MCs, (**f**) heating–cooling cycle stability of *x* = 0.10 MCs.

The storage stability is evaluated for the samples with nominal Zn fractions of *x* = 0, 0.05, and 0.10. Long-term storage measurements were focused on *x* ≤ 0.10, with *x* = 0.10 selected as the representative Zn-containing composition for subsequent stability and device evaluations. Therefore, the *x* = 0.10 sample is selected as the representative Zn-containing composition because it provides a favorable balance between luminescence efficiency and storage stability. Figure 4a and Figure S2 present the storage stability of TEA_2_Mn_1−*x*_Zn*_x_*Br_4_ MCs as a function of Zn^2+^ substitution level. After 60 days of storage, the PL intensity of the unsubstituted TEA_2_MnBr_4_ MCs decreases by 14.1 ± 0.8%, while those of *x* = 0.05 MCs and *x* = 0.10 MCs drop by 12.8 ± 0.5% and 6.2 ± 0.6%, respectively. These results indicate that Zn^2+^ substitution can enhance the storage stability of the MCs. The TG curve of *x* = 0.00 MCs and *x* = 0.10 MCs, shown in Figure 4b,c, reveals that notable weight loss occurs only at 222 °C and 225 °C, respectively. In addition, the temperature-dependent PL emission pseudocolor contour maps of *x* = 0.10 MCs are shown in Figure 4d. With the increase in temperature, the green PL intensity of TEA_2_MnBr_4_ and *x* = 0.10 MCs gradually declines (Figure S3a). At 393 K, the *x* = 0.10 MCs retain 60.3% of their initial PL intensity, whereas the *x* = 0.05 MCs retain 53.6%. The activation energies (ΔE) of *x* = 0.05 MCs and *x* = 0.10 MCs are calculated by Arrhenius equation:

*I*_(*T*)_ = *I*_0_/[1 + A exp (−ΔE∕kT)] (3)

where A is a constant, *k* is the Boltzmann constant, *I*_0_ is the PL intensity at 293 K, and *I*_(T)_ is the PL intensity at temperature T. Arrhenius fitting of the temperature-dependent PL intensity gives ΔE of 0.2719 ± 0.011 eV for *x* = 0.05 MCs and 0.2866 ± 0.026 eV for *x* = 0.10 MCs (Figure S3b).

To distinguish reversible thermal quenching from irreversible thermal degradation, repeated heating–cooling PL measurements were performed between 298 and 413 K. As shown in Figure 4f, the normalized PL intensity decreases to about 47 % upon heating to 413 K but recovers to nearly its initial value after cooling back to 298 K. The heating and cooling spectra collected over four consecutive cycles exhibit almost unchanged emission peak positions and spectral profiles (Figure S4). These cycling results indicate that the thermal quenching of the *x* = 0.10 MCs is largely reversible under the present conditions. Together with the comparative storage and temperature-dependent PL measurements of the *x* = 0.00 and *x* = 0.10 samples, the results indicate improved storage and thermal stability after Zn^2+^ substitution.

**Figure 5:**
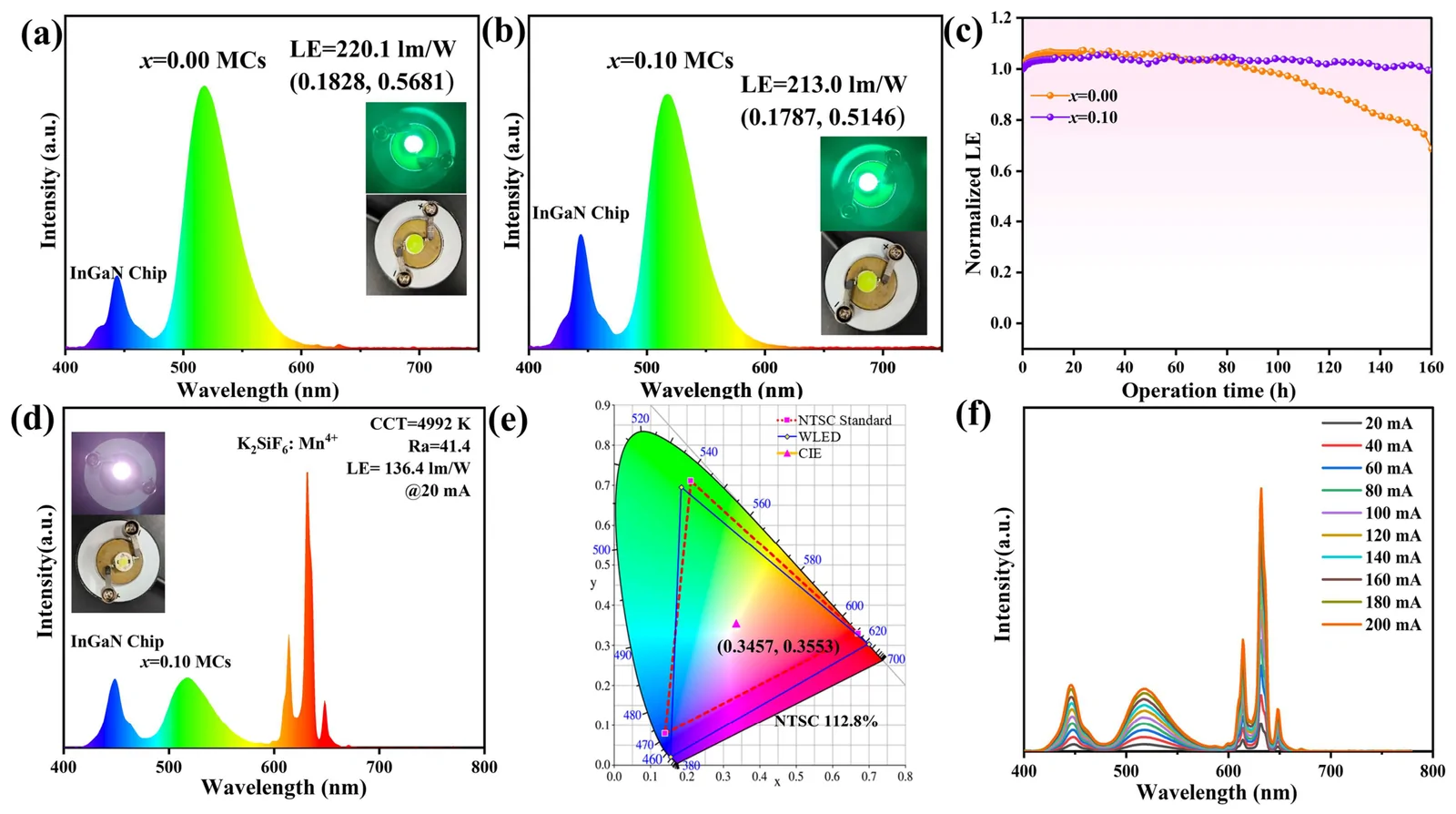
EL spectra of green LED devices based on (**a**) *x* = 0.00 and (**b**) *x* = 0.10 MCs. The insets in panels (**a**,**b**) show photographs of the corresponding green LED devices operated at 20 mA. (**c**) Operational stability of green LED devices. (**d**) The EL spectrum of WLED devices based on the *x* = 0.10 MCs and K_2_SiF_6_:Mn^4+^. The inset photographs are corresponding WLED devices operated at 20 mA. (**e**) CIE coordinates and (**f**) EL spectra of WLEDs with different currents.

To evaluate practical potential, the *x* = 0.00 MCs and *x* = 0.10 MCs were employed as light-conversion materials in LED devices. Figure 5a,b present the EL spectra of green LEDs fabricated using *x* = 0.00 and *x* = 0.10 MCs, respectively. The fabricated green LED based on *x* = 0.00 MCs exhibits green emission with Commission Internationale de l’Éclairage (CIE) chromaticity coordinates of (0.1828, 0.5681), while the green LEDs based on *x*=0.10 MCs have CIE coordinates of (0.1787, 0.5146). At a driving current of 20 mA, the corresponding LEs reach about 220.1 lm/W and 213.0 lm/W. As shown in Figure 5c, the operational stability of the green LED devices was evaluated using two devices for each composition (*n* = 2) under identical conditions. Both devices were continuously driven at 20 mA for 160 h at 25 °C and 50% relative humidity. As shown in Figure 5c, the *x* = 0.00 MCs-based devices can retain 68.7 ± 0.2% of their initial LE after continuous operation for 160 h. In contrast, the *x* = 0.10 MCs-based devices can maintain 99.4 ± 0.1%. It should be noted that the reported 3000 h result refers to the spectral and chromaticity stability of a WLED [14], whereas the present operational-stability test evaluates the luminous-efficacy retention of green LED devices; therefore, the two results should not be directly compared as equivalent device-lifetime measurements. The EL spectra and color stability were also monitored during continuous operation. As shown in Figure S5, the green EL intensity of the unsubstituted device decreases by 26.3 ± 0.8%, whereas the spectral profile and intensity of the *x* = 0.10 MCs-based devices remain nearly unchanged after 160 h. The dominant emission wavelengths of both devices remain at approximately 518 nm throughout the test. In addition, the CIE coordinates of the *x* = 0.10 MCs-based device exhibit only minor variations, indicating good color stability during continuous operation. The enhanced stability may be associated with Zn^2+^-induced lattice contraction and increased thermal activation energy [24].

Although *x* = 0.05 MCs show a slightly higher PLQY, *x* = 0.10 MCs provide a better balance between luminescence efficiency, storage stability, and device operational stability, and were therefore selected for LED fabrication. A white LED device was fabricated using the x = 0.10 MCs and K_2_SiF_6_:Mn^4+^. Figure 5d shows the EL spectrum of a white LED. Under a driving current of 20 mA, the white LED exhibits CIE chromaticity coordinates of (0.3457, 0.3553), close to the equal-energy white point E(1/3, 1/3). The white LED has a CRI of about 41.4 and an LE of about 136.4 lm/W with a CCT of about 4990 K. The relatively low CRI indicates that the white LED is appropriate for wide-gamut display-backlight applications, rather than for general illumination requiring high color-rendering quality. The calculated color gamut covers about 112.8% in the CIE 1931 *x*, *y* chromaticity space, indicating that the *x* = 0.10 MCs have broad application prospects in wide-gamut displays. Furthermore, the electroluminescence spectral profile remains unchanged with increasing drive current (Figure 5f), while the EL intensity progressively increases. These results demonstrate the excellent stability of the *x* = 0.10 MCs-based LED devices.

## 4. Conclusions

In summary, Zn^2+^-substituted TEA_2_MnBr_4_ MCs were successfully synthesized through a facile ultrafast self-assembly method. With the increase in Zn^2+^, the PL intensity of MCs gradually decreases. The *x* = 0.10 MCs exhibit green emission at 518 nm with a narrow FWHM of 43.8 nm and a PLQY of about 85%. Compared with the *x* = 0.00 MCs, the *x* = 0.10 MCs show improved storage and thermal stability, retaining above 93% of their initial PL intensity after 60 days of storage. The corresponding green LEDs can achieve a high LE of about 213.0 lm/W and retain above 99% of their initial luminous efficacy after continuous operation at 20 mA for 160 h. A white LED fabricated by combining *x* = 0.10 MCs with K_2_SiF_6_:Mn^4+^ red phosphors achieves a high LE of about 136.4 lm/W, and a wide color gamut covering 112.8 % of the NTSC 1931. These results demonstrate that Zn^2+^ substitution is an effective strategy for improving the stability of hybrid manganese bromide emitters and highlight the potential of TEA_2_ Mn_1−*x*_Zn*_x_*Br_4_ MCs for stable, lead-free, wide-gamut display applications.

## Data Availability

The data presented in this study are available on request from the corresponding authors.

## Supplementary Materials

The following supporting information can be downloaded at: https://www.mdpi.com/article/10.3390/ma19173650/s1, Figure S1: Rietveld refinement profiles of TEA_2_Mn_1-*x*_Zn*_x_*Br_4_ MCs with nominal Zn fractions of (a) *x* = 0.00, (b) *x* = 0.10, and (c) *x* = 0.25. (d) Refined lattice parameters *a* and *c*, and unit-cell volume V, as functions of nominal Zn content; Figure S2: Storage stability of TEA_2_Mn_1-*x*_Zn*_x_*Br_4_ MCs with different Zn^2+^ substitution contents: (a) *x* = 0.00, (b) *x* = 0.05, and (c) *x* = 0.10; Figure S3: (a) Temperature-dependent PL emission pseudocolor contour maps of *x* = 0.00 MCs, (b) thermal activation energy of *x* = 0.00 and *x* = 0.10 MCs; Figure S4: (a) PL spectra recorded after cooling back to 298 K following each heating cycle of *x* = 0.10 MCs, (b) PL spectra collected at 413 K during four consecutive heating cycles of *x* = 0.10 MCs; Figure S5. EL spectra of the (a) *x* = 0.00 and (b) *x* = 0.10 MCs–based LEDs measured at 1 min and 160 h, (c) dominant emission wavelength, (d) normalized green–emission intensity, (e) CIE coordinates as a function of operation time, and (f) CIE–coordinate changes before and after 160 h of continuous operation; Table S1: The nominal compositions of TEA_2_Mn_1-*x*_Zn*_x_*Br_4_; Table S2: Rietveld refinement results of TEA_2_Mn_1-*x*_Zn*_x_*Br_4_ MCs with different Zn^2+^ substitution amounts; Table S3: Elemental analysis of TEA_2_Mn_1-*x*_Zn*_x_*Br_4_ MCs based on EDS results; Table S4: Elemental analysis of TEA_2_Mn_1-*x*_Zn*_x_*Br_4_ MCs based on XPS results; Table S5: PLQY, *η*_ab,_ and EQE of TEA_2_Mn_1-*x*_Zn*_x_*Br_4_ MCs.
