# Supplementary material for: Boosting Stability of Hybrid Manganese Bromide Microcrystals by Zn2+ Substitution for Wide-Gamut Displays
Source: Materials (Basel). 2026 Aug 27;19(17):3650. doi: 10.3390/ma19173650 (PMC13567270; doi:10.3390/ma19173650)
Supplement: Supplementary file 1 [file materials-19-03650-s001.zip › materials-4441202_SpM.pdf]

Boosting Stability of Hybrid Manganese Bromide Microcrystals by Zn^{2+} Substitution for Wide Gamut Displays

Huidong Tang[1, *], Pengcheng Jiang[1], Xinyi Wen[1], Yulu He[1], Simeng Wu[1], Xin Xiong[1], Yanqiao Xu[2], Zhi Wu[1, *], Qing Hu[3, *]

1 School of Material Science and Engineering, Hunan Institute of Technology, Hengyang 421002, P. R. China

2 National Engineering Research Center for Domestic & Building Ceramics, Jingdezhen Ceramic University, Jingdezhen 333001, P. R. China

3 School of Material Science and Engineering, Jingdezhen Ceramic University, Jingdezhen 333001, P. R. China

*Authors to whom correspondence should be addressed.

E-mail: tanghuidong@hnit.edu.cn, 2017002025@hnit.edu.cn, huqing@jci.edu.cn

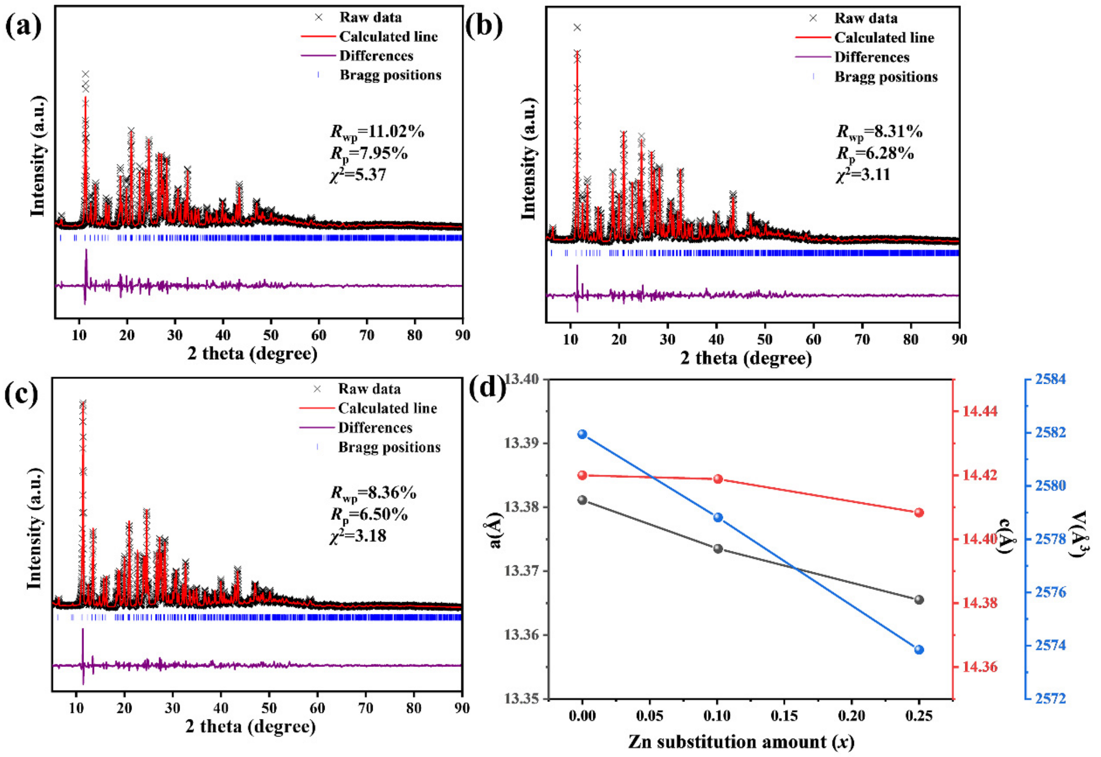

Figure S1. Rietveld refinement profiles of $TEA_2Mn_{1-x}Zn_xBr_4$ MCs with nominal Zn fractions of (a) x=0.00, (b) x=0.10, and (c) x=0.25. (d) Refined lattice parameters a and c, and unit-cell volume V, as functions of nominal Zn content.

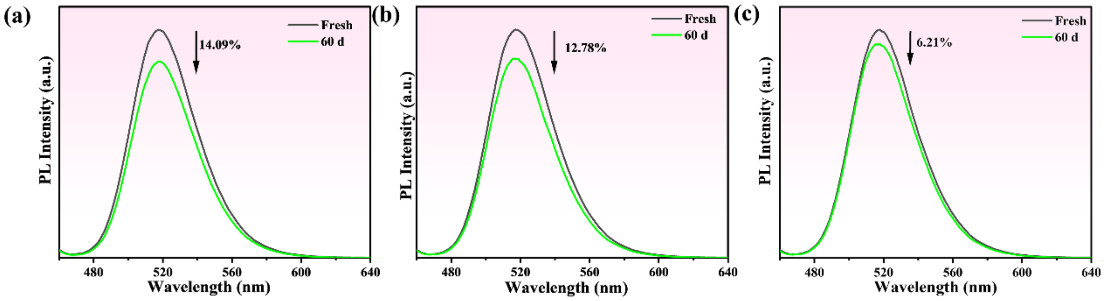

Figure S2. Storage stability of $TEA_2Mn_{1-x}Zn_xBr_4$ MCs with different Zn^{2+} substitution contents: (a) x=0.00, (b) x=0.05, and (c) x=0.10.

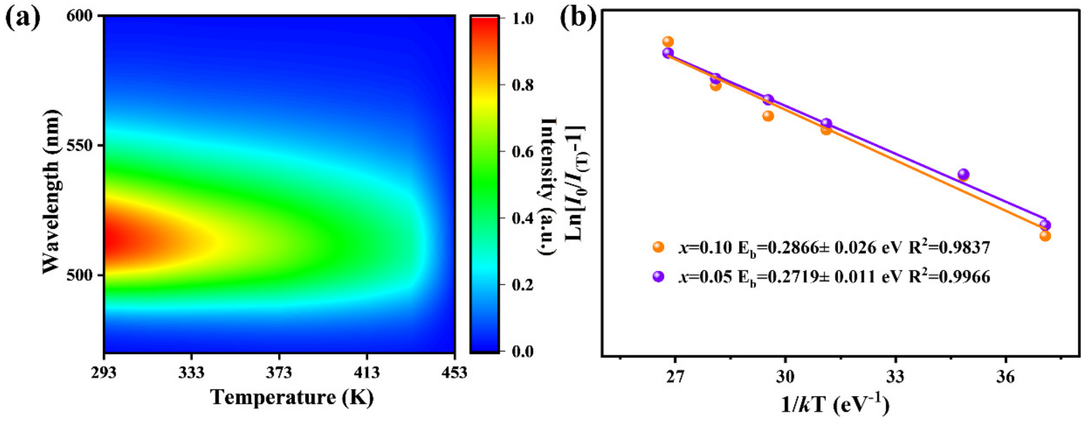

Figure S3. (a) Temperature-dependent PL emission pseudocolor contour maps of x=0.05 MCs, (b) thermal activation energy of x=0.05 and x=0.10 MCs.

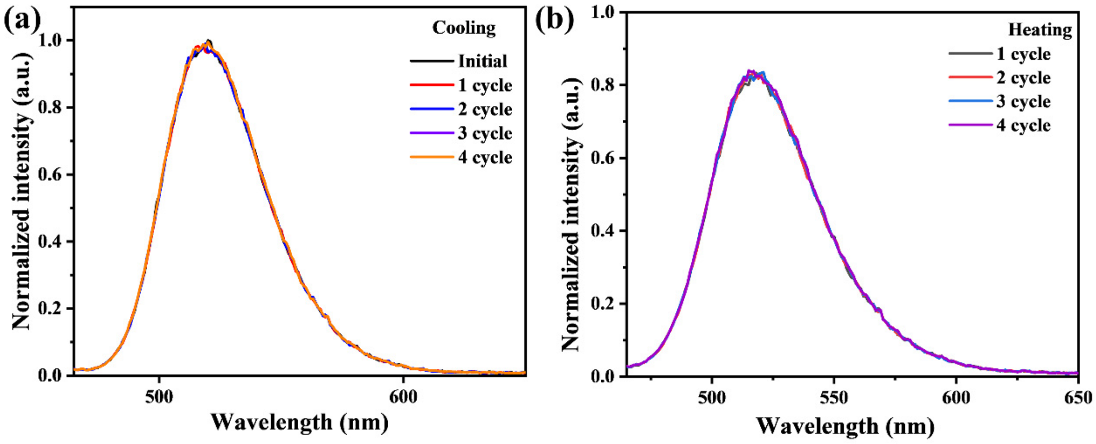

Figure S4. (a) PL spectra recorded after cooling back to 298 K following each heating cycle of x=0.10 MCs, (b) PL spectra collected at 413 K during four consecutive heating cycles of x=0.10 MCs.

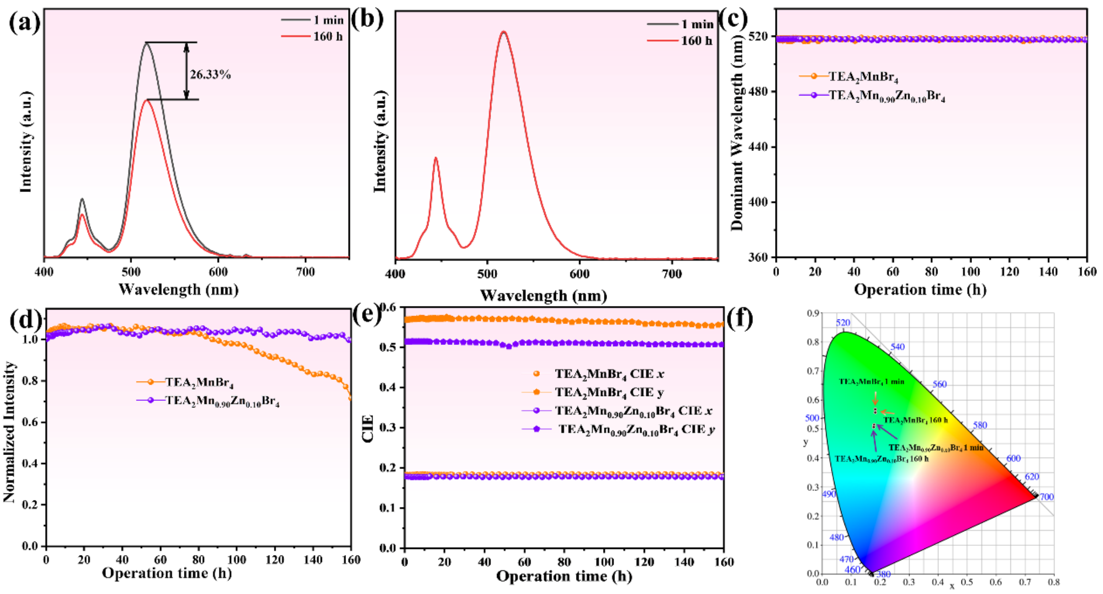

Figure S5. EL spectra of the (a) x=0.00 and (b) x=0.10 MCs–based LEDs measured at 1 min and 160 h, (c) dominant emission wavelength, (d) normalized green–emission intensity, (e) CIE coordinates as a function of operation time, and (f) CIE–coordinate changes before and after 160 h of continuous operation.

Table S1. The nominal compositions of $TEA_2Mn_{1-x}Zn_xBr_4$.

$TEA_2Mn_{1-x}Zn_xBr_4$ Sample	x	TEABr	$MnBr_2{\cdot}4H_2O$	$ZnBr_2$	C_2H_5OH
TEA_2MnBr_4	0.00	2.25 mmol	1.00 mmol	0.00 mmol	10 mL
$TEA_2Mn_{0.95}Zn_{0.05}Br_4$	0.05	2.25 mmol	0.95 mmol	0.05 mmol	10 mL
$TEA_2Mn_{0.90}Zn_{0.10}Br_4$	0.10	2.25 mmol	0.90 mmol	0.10 mmol	10 mL
$TEA_2Mn_{0.85}Zn_{0.15}Br_4$	0.15	2.25 mmol	0.85 mmol	0.15 mmol	10 mL
$TEA_2Mn_{0.80}Zn_{0.20}Br_4$	0.20	2.25 mmol	0.80 mmol	0.20 mmol	10 mL
$TEA_2Mn_{0.75}Zn_{0.25}Br_4$	0.25	2.25 mmol	0.75 mmol	0.25 mmol	10 mL

Table S2. Rietveld refinement results of $TEA_2Mn_{1-x}Zn_xBr_4$ MCs with different Zn^{2+} substitution amounts.

Zn substitution amount (x)	a= b (Å)	c(Å)	V(Å^3)	R_{wp}	R_p	χ^2
x =0.00	13.3810(4)	14.4200(4)	2581.92(16)	11.02%	7.95%	5.37
x =0.10	13.3735(3)	14.4188(3)	2578.81(11)	8.31%	6.28%	3.11
x =0.25	13.3655(2)	14.4083(4)	2573.84(11)	8.36%	6.50%	3.18

Table S3. Elemental analysis of $TEA_2Mn_{1-x}Zn_xBr_4$ MCs based on EDS results.

$TEA_2Mn_{1-x}Zn_xBr_4$	N Atomic (%)	Mn Atomic (%)	Zn Atomic (%)	Br Atomic (%)	Zn/(Zn+Mn) (%)
x =0.00	31.4	12.6	0	56.0	0
x =0.10	36.4	11.2	1.2	51.1	9.7

Table S4. Elemental analysis of $TEA_2Mn_{1-x}Zn_xBr_4$ MCs based on XPS results.

$TEA_2Mn_{1-x}Zn_xBr_4$	Mn Atomic (%)	Zn Atomic (%)	Br Atomic (%)	Zn/(Zn+Mn) (%)
x =0.00	1.79	0	8.53	0
x =0.10	0.98	0.10	9.08	9.3

Table S5. PLQY, η_{ab}, and EQE of $TEA_2Mn_{1-x}Zn_xBr_4$ MCs.

$TEA_2Mn_{1-x}Zn_xBr_4$	PLQY(%)	η_{ab}(%)	EQE(%)
x =0.00	98.1 ± 0.6	45.0 ± 0.7	44.1 ± 0.7
x =0.05	91.4 ± 0.8	62.0 ± 1.0	56.7 ±1.0
x =0.10	85.6 ± 0.6	65.0 ± 1.1	55.6 ±1.0
x =0.25	78.3 ± 0.9	43.0 ± 0.6	33.7 ± 0.6
